# Deleterious alterations in homologous recombination repair genes and efficacy of platinum-based chemotherapy in biliary tract cancers

**DOI:** 10.1093/oncolo/oyae125

**Published:** 2024-06-01

**Authors:** Carmen Belli, Luca Boscolo Bielo, Matteo Repetto, Edoardo Crimini, Raimondo Scalia, Anna Diana, Jessica Orefice, Liliana Ascione, Gloria Pellizzari, Nicola Fusco, Massimo Barberis, Bruno Daniele, Elena Guerini-Rocco, Giuseppe Curigliano

**Affiliations:** Division of New Drugs and Early Drug Development for Innovative Therapies, European Institute of Oncology, IRCCS, Milan 20141, Italy; Division of New Drugs and Early Drug Development for Innovative Therapies, European Institute of Oncology, IRCCS, Milan 20141, Italy; Department of Oncology and Hemato-Oncology, University of Milan, Milan 20122, Italy; Division of New Drugs and Early Drug Development for Innovative Therapies, European Institute of Oncology, IRCCS, Milan 20141, Italy; Department of Oncology and Hemato-Oncology, University of Milan, Milan 20122, Italy; Early Drug Development Service, Memorial Sloan-Kettering Cancer Center, New York 10065, United States; Division of New Drugs and Early Drug Development for Innovative Therapies, European Institute of Oncology, IRCCS, Milan 20141, Italy; Department of Oncology and Hemato-Oncology, University of Milan, Milan 20122, Italy; Division of New Drugs and Early Drug Development for Innovative Therapies, European Institute of Oncology, IRCCS, Milan 20141, Italy; Medical Oncology Unit, Ospedale del Mare, Naples 80147, Italy; Medical Oncology Unit, Ospedale del Mare, Naples 80147, Italy; Division of New Drugs and Early Drug Development for Innovative Therapies, European Institute of Oncology, IRCCS, Milan 20141, Italy; Department of Oncology and Hemato-Oncology, University of Milan, Milan 20122, Italy; Division of New Drugs and Early Drug Development for Innovative Therapies, European Institute of Oncology, IRCCS, Milan 20141, Italy; Department of Oncology and Hemato-Oncology, University of Milan, Milan 20122, Italy; Department of Oncology and Hemato-Oncology, University of Milan, Milan 20122, Italy; Division of Pathology, IEO, European Institute of Oncology IRCCS, Milan 20141, Italy; Division of Pathology, IEO, European Institute of Oncology IRCCS, Milan 20141, Italy; Medical Oncology Unit, Ospedale del Mare, Naples 80147, Italy; Department of Oncology and Hemato-Oncology, University of Milan, Milan 20122, Italy; Division of Pathology, IEO, European Institute of Oncology IRCCS, Milan 20141, Italy; Division of New Drugs and Early Drug Development for Innovative Therapies, European Institute of Oncology, IRCCS, Milan 20141, Italy; Department of Oncology and Hemato-Oncology, University of Milan, Milan 20122, Italy

**Keywords:** biliary tract cancer, recombination, DNA damage response, platinum agents, chemotherapy

## Abstract

**Background:**

Platinum-based chemotherapy represents the standard first-line treatment for biliary tract cancers (BTC). Deficits in genes involved in the homologous recombination (HR) and DNA damage response (DDR) may confer higher sensitivity to platinum agents.

**Methods:**

We retrospectively included patients affected by BTC from 2 Italian institutions. Inclusion criteria consist of the receipt of platinum-based chemotherapy in the metastatic setting and the availability of comprehensive genomic profiling using next-generation sequencing (NGS). Patients were included in the HRD-like group if demonstrated oncogenic or likely oncogenic alterations in HR-/DDR-genes. Clinical endpoints were compared between the HRD-like group and the non-HRD-like group.

**Results:**

Seventy-four patients were included, of whom 25 (33%) in the HRD-like group and 49 (66%) in the non-HRD group. With a median follow-up of 26.04 months (interquartile-range [IQR] 9.41-29.27) in the HRD-like group and of 22.48 months (IQR 16.86-40.53) in the non-HRD group, no PFS difference emerged, with a mPFS of 5.18 months in the HRD-like group compared to 6.04 months in the non-HRD group (hazard ratio [HR], 1.017, 95% CI 0.58-1.78; *P* = .95). No differences were observed in DCR (64% [95 CI 45%-83%] vs 73% [95 CI 61%-86%]; *P* = .4), and CBR (45% [95% CI 28%-73%] vs 50% [95% CI, 37%-68%]; *P* = .9) between the HRD-like group and non-HRD groups, respectively. Median OS did not statistically differ between the HRD-like group and non-HRD group (26.7 vs 18.0 months, respectively; HR, 0.670, 0.33 to 1.37, *P* = .27).

**Conclusion:**

HR-/DDR-genes, when assessed with regular tumor-only NGS panels, provide limited clinical validity to identify patients with BTC more likely to benefit from platinum-based chemotherapy.

## Teaser

This study, including biliary tract cancers, investigated and demonstrated the lack of predictive value of alterations occurring HR-/DDR-genes to predict the efficacy of platinum-based chemotherapy, suggesting the limited analytical validity of assessing the HRD-signature with regular tumor-only NGS panels.

**Implications for practice**

This study explored whether alterations in HR-/DDR-genes could serve as predictors of the efficacy of platinum-based chemotherapy in biliary tract cancers (BTC), and no difference were observed between HRD-like and non-HRD tumors. Consequently, results provided evidence concerning the limited clinical validity of HR-/DDR-gene alterations to predict platinum efficacy in BTC when assessed with conventional tumor-only NGS panels. Accordingly, this research underscores the importance of integrating diagnostic methods to achieve higher analytical validity to define the HRD- signature, particularly among non-BRCA1/2 associated tumors. Moreover, the study may underscore the rationale for testing HRD-genomic signature also beyond BRCA1/2 associated tumors, to refine the selection of patients more likely to benefit from DNA-damaging agents to achieve treatment personalization, additional treatment opportunities and ultimately improved patient-centric clinical outcomes.

## Introduction

Biliary tract cancers (BTCs) represent a heterogeneous group of tumors, sharing a poor prognosis due to inadequate early detection tools, difficult anatomical access, and aggressive tumor biology.^1,2^

Platinum-based chemotherapy still represents the standard first-line treatment for BTC in a one-size-fits-all approach.^3-7^ The combination of durvalumab, gemcitabine, and cisplatin recently emerged as the optimal first-line therapy for advanced BTC as a result of the TOPAZ-1 trial.^8^ Moreover, the KEYNOTE-966 trial showed encouraging results for the combination of pembrolizumab with gemcitabine and cisplatin in a similar context.^9^ Despite immunotherapy yielding new therapeutic options for BTC, median overall survival from the use of front-line platinum regimens still approximates 1 year.^1,2^ Moreover, an increasing number of actionable genomic alterations recently emerged as a therapeutic option for BTC, accounting for approximately 40% of cases,^10-12^ whose presence challenges the current one-size-fits-all approach for treating patients with upfront platinum-based chemotherapy, demanding predictive biomarkers to adapt precision medicine also as a front-line strategy in treating such tumors.

Targeting the homologous recombination (HR) and DNA damage repair (DDR) pathways have been revealed in recent years as one of the most promising treatment strategies, whose deficits frequently occur across solid tumors.^13-15^ Among 17 000 solid tumors subjected to high-throughput genetic profiling, alterations in HR-/DDR-genes were reported in 17.4% of cancers, with the highest frequencies reported in endometrial cancer (34.4%), biliary tract (28.9%), bladder (23.9%), hepatocellular (20.9%), and ovarian cancer (20.0%). In the same study, ARID1A (7.2%), BRCA2 (3.0%), BRCA1 (2.8%), ATM (1.3%), ATRX (1.3%), and CHEK2 (1.3%) were the HR-/DDR-genes most commonly mutated.^13^

While BRCA1/2 are sensitive indicators of HRD, other biomarkers such as alterations in other HR-/DDR-genes have been proposed to identify tumors more likely to benefit from platinum-based therapies and poly-ADP ribose polymerase (PARP) inhibitors (PARPi).^16-19^ As such, expanding the cohort of patients who may benefit from therapies targeting the HR-/DDR-pathway still represents an unmet need in current clinical practice to further optimize clinical outcomes of patients affected by solid tumors.

Considering these elements, we performed a retrospective analysis to investigate whether alterations in genes involved in the HR-/DDR-pathway may relate to patients’ clinical outcomes from platinum-based chemotherapy in BTC.

## Methods

### Patients selection

We conducted a retrospective study of patients affected by biliary tract cancer (BTC) from 2 Italian institutions (European Institute of Oncology, Milan; Hospital of the Sea, Naples). Inclusion criteria consisted of the receipt of platinum-based chemotherapy for the metastatic disease, for which solid- or blood-based Next-Generation Sequencing reports were available (FoundationOne CDx, FoundationOne Liquid CDx, Oncomine Comprehensive Assay v3). Clinical data were extracted from electronic medical reports with a follow-up cutoff established in July 2023.

Patients were allocated in the HRD-like and non-HRD groups according to the presence of oncogenic and likely oncogenic alterations in at least 1 HR-/DDR-gene, including ARID1A, ATM, ATRX, BAP1, BARD1, BLM, BRCA1/2, BRIP1, CHEK1/2, and FANCA/C/D2/E/F/G/L, PALB2, RAD50, RAD51, RAD51B according to previously reported studies.^13^ Genomic alterations were annotated based on the initial NGS report and subsequently reviewed using OncoKB.^20^ Alterations were deemed oncogenic or likely oncogenic if annotated as such in at least 1 annotation resource.

The research was conducted in accordance with the principles stated in the Declaration of Helsinki and with the principles of good clinical practice.

### Endpoints and statistical analysis

Clinical outcomes of interest in the study included median progression-free survival (mPFS), defined from the start of the first platinum-based chemotherapy received in the metastatic setting to disease progression or death, whichever occurred first; median overall survival (mOS), calculated as the time from the start of platinum-based chemotherapy in the metastatic setting to death; disease control rate (DCR), defined by the sum of stable disease, partial response and complete response at the first radiological reevaluation during the receipt of platinum-based chemotherapy; and clinical benefit rate (CBR), defined as the percentage of patients showing disease control, partial response, or complete response for at least 6 months from the start of platinum-based chemotherapy. DCR was preferred over the overall response rate as no reevaluation according to RECIST 1.1 criteria was performed for patients not showing disease progression during radiological monitoring.

Continuous variables were reported as median and range or mean and standard error, as appropriate, and compared between groups using the Mann–Whitney test. Categorical variables were expressed as numbers and proportions and compared using Fisher’s exact test.

Median follow-up time was calculated using the reverse Kaplan-Meier method for each HRD-like group and non-HRD-group.

Kaplan-Meier method was used to estimate medians and time-to-event endpoints between the HRD-like group and the non-HRD group were compared using log-rank statistics to account for censored data. The Cox proportional hazard model was used to estimate hazard ratios and 95% confidence intervals (CI).

Non-adjusted hazard ratios to assess the association between baseline clinicopathological characteristics with PFS and OS were calculated using the univariable Cox regression model and used to adjust the main effect of the HRD-status in the multivariable model. Variables were included as covariates in the Cox regression model if demonstrated to satisfy the proportional hazards assumption. If the Cox regression assumption was not met for a clinically meaningful variable, the latter was used as a stratification variable in the Cox model. All tests were performed assuming a 2-sided statistical significance with an alpha level of <.05.

Statistical analyses were performed using R software version 4.2.2.^21^

## Results

### Patient characteristics

Seventy-four consecutive patients treated across the 2 Institutions between October 2016 and May 2023 were included in the study. Of these, 25 were included in the HRD-like group and 49 in the non-HRD-like group. Patients’ baseline characteristics were evenly distributed between the 2 groups as reported in Table 1.

**Table 1.** Baseline patient’s characteristics.

Groups	HRD, *N* = 25^a^	Non-HRD, *N* = 49^a^	*P* value^b^
Age, median (range)	66 (45-77)	65 (39-81)	.7
Sex			.3
Female	15/ 25 (60%)	23/ 49 (47%)	
Male	10/ 25 (40%)	26/ 49 (53%)	
Primary tumor type			.2
Extrahepatic	4/ 25 (16%)	14/ 49 (29%)	
Gallbladder	3/ 25 (12%)	11/ 49 (22%)	
Intrahepatic	18/ 25 (72%)	24/ 49 (49%)	
Nr. metastatic sites			.4
≤2	15/ 25 (60%)	34/ 49 (69%)	
≥3	10/ 25 (40%)	15/ 49 (31%)	
De novo stage IV	15/ 25 (60%)	30/ 49 (61%)	>.9
Previous (neo)adjuvant chemotherapy	7/ 25 (28%)	16/ 49 (33%)	.7
Line first platinum chemotherapy			>.9
≥2 L	2/ 25 (8.0%)	3/ 49 (6.1%)	
1 L	23/ 25 (92%)	46/ 49 (94%)	

^a^Median (minimum-maximum); *n*/*N* (%).

^b^Wilcoxon rank sum test; Pearson’s Chi-squared test; Fisher’s exact test.

### Genomic alterations

NGS was performed on tissue biopsy in 92% of the patients and liquid biopsy in 8.1% of the cases. In the former group, biopsies were performed on the primary tumor site in 84% of the cases, and on the metastatic site in 16%, which included lymph nodes (*n* = 2), ovary (*n* = 1), peritoneum (*n* = 2), kidney (*n* = 1), and lung (*n* = 1). Tumor mutational burden (TMB) was available for 45 patients. Median TMB was 4.12 ± 3.8, with a high TMB (≥10 mutations/mega base) found in 5 patients (11.1%). No difference in TMB was observed between the HRD-like group (4.7 ± 3.9) and the non-HRD-group (3.5 ± 3.8) (*P* = .07). Among 53 patients assessed for microsatellite instability, high microsatellite instability (MSI-H) was found in 3 patients (5.6%).

**Figure 1.**
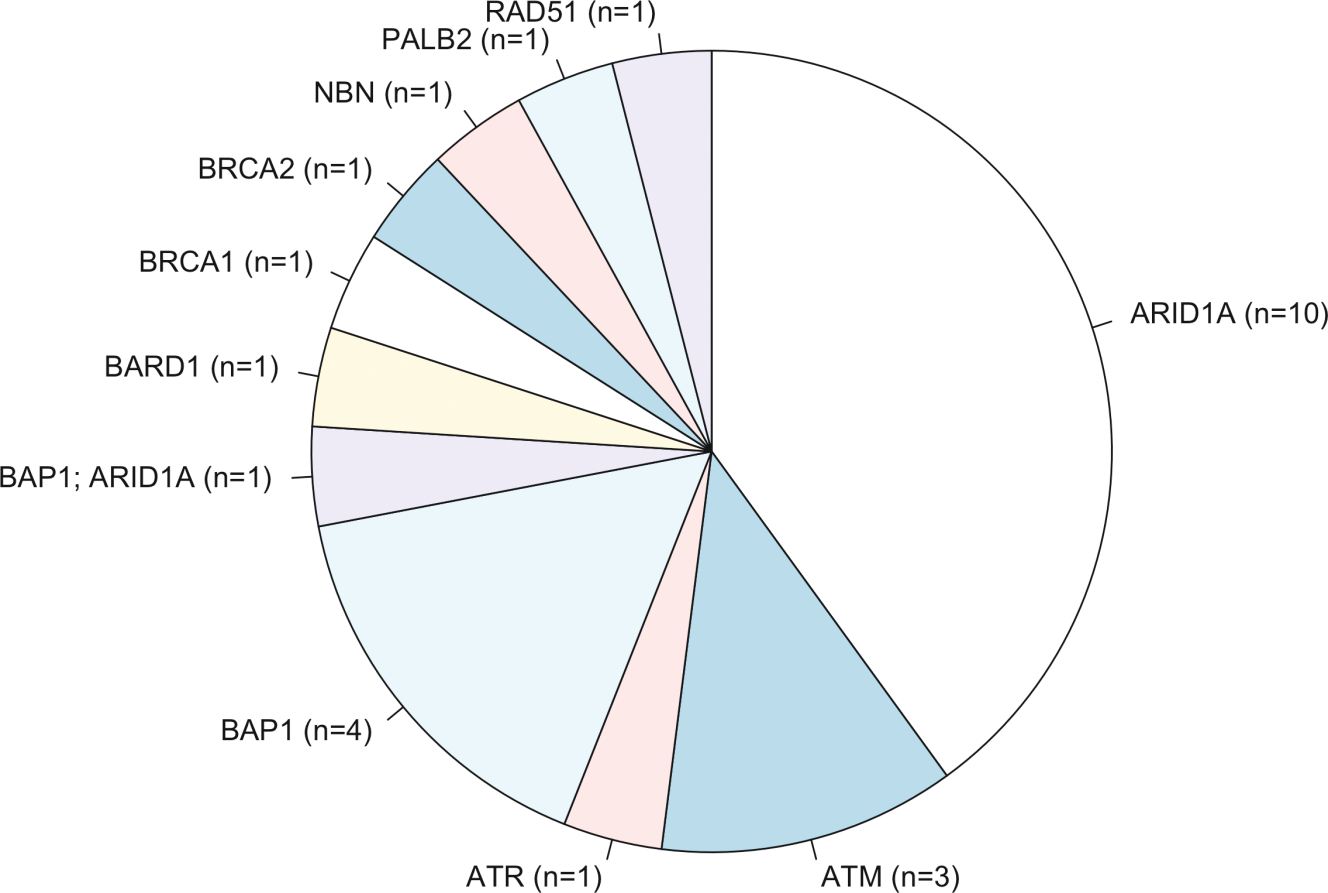
HR-/DDR-genes alterations. Distribution of HR-/DDR-related gene alterations in the HRD-like group (*n* = 25). Keys: *n*, number.

Among the 25 patients in the HRD-like group, the most common altered HR-/DDR-gene was ARID1A (*n* = 10, 40%), followed by BAP1 (*n* = 4, 16%), and ATM (*n* = 3, 12%) (Figure 1).

**Figure 2.**
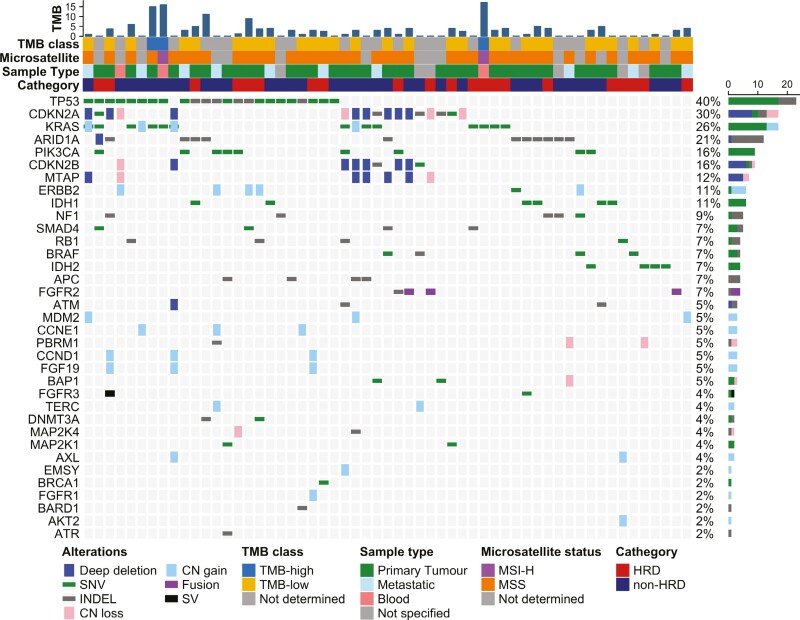
Genomic alterations among HRD-like and non-HRD tumors. Oncoprint of most common oncogenic and likely oncogenic alterations observed in the whole cohort of tumors, classified for the class of genomic alterations and baseline clinicopathological variables. Abbreviations: SNV, single nucleotide variant; INDEL, insertion–deletion; CN loss, copy number loss; CN gain, copy number gain; SV, structural variant; TMB, tumor mutational burden; MSI-H, high microsatellite instability; MSS, microsatellite stability.

The most commonly encountered oncogenic and likely oncogenic alterations in the whole cohort of patients are reported in Figure 2.

### Clinical outcomes

In the HRD-like group, 24% (*n* = 6) of patients received previous adjuvant chemotherapy in the early setting of the disease, of whom 5 patients received capecitabine and 1 patient gemcitabine. In the non-HRD group, 30.6% (*n* = 15) of patients received neo(adjuvant) chemotherapy, with 12 patients receiving adjuvant chemotherapy (*n* = 5 capecitabine, *n* = 2 cisplatin plus gemcitabine, and *n* = 5 gemcitabine) and 3 patients neoadjuvant chemotherapy (*n* = 1 cisplatin plus gemcitabine and *n* = 2 nab-paclitaxel plus gemcitabine). All patients receiving neo(adjuvant) chemotherapy underwent curative-intent surgery, with 2 patients receiving adjuvant radiotherapy (both in the non-HRD group).

**Figure 3.**
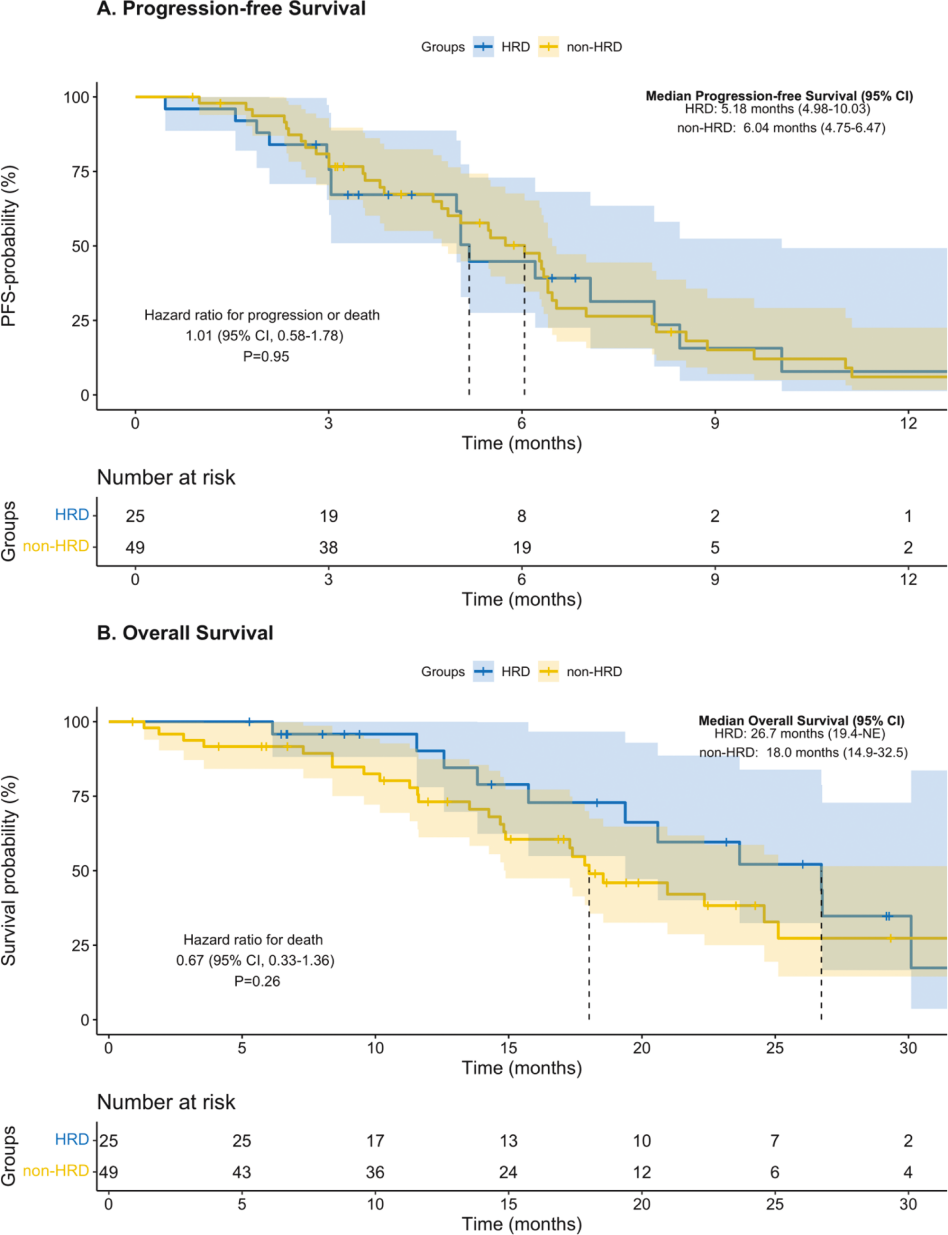
Survival analysis in HRD-like and non-HRD group. Kaplan–Meier plots of progression-free survival (A) and overall survival (B) among HRD-like tumors and non-HRD tumors.

Among the 2 groups, 92% (*n* = 23) of patients in the HRD-like group and 94% (*n* = 46) in the non-HRD group received platinum-based chemotherapy in the first-line setting. Most patients in the HRD-like group (76%, *n* = 19) and non-HRD group (67%, *n* = 33) received cisplatin plus gemcitabine as the first-line regimen, with only 2 patients in the HRD-like group and 3 patients in the non-HRD group receiving the combination of cisplatin, gemcitabine, and durvalumab. With a median follow-up of 26.04 months (IQR 9.41-29.27) in the HRD-like group and 22.48 months (IQR 16.86-40.53) in the non-HRD group, PFS events were recorded in 72% (18 of 25) and 81.63% (40 of 49) of patients in the HRD-like and non-HRD group, respectively. No difference in mPFS emerged between the HRD-like group (5.18 months; 95% CI, 4.98-10.03) and non-HRD group (6.04 months; 95% CI, 4.75-6.47) (hazard ratio (HR) 1.017; 95% CI, 0.58-1.78, *P* = .95) (Figure 3).

A DCR of 64.0% (16 of 25, [95 CI 45%-83%]) in the HRD-like group and of 73.5% (36 of 49, [95 CI 61%-86%]) in the non-HRD group was observed (OR 1.54 [95% CI 0.47-4.90]; *P* = .4), with an overall CBR of 45% (95% CI, 28%-73%) in the HRD-like group, compared to 50% (95% CI, 37%-68%) in the non-HRD group (*P* = .9).

**Figure 4.**
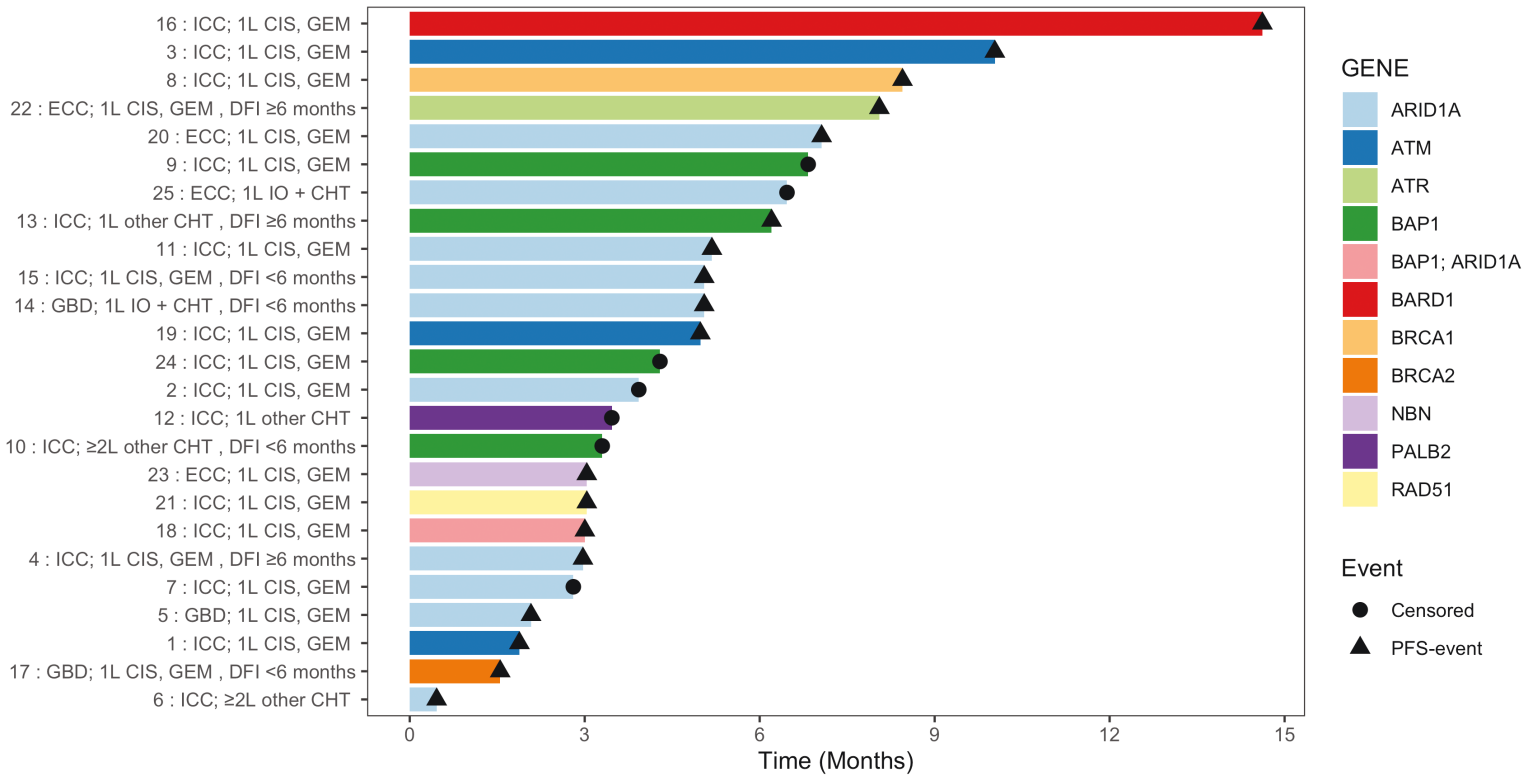
HR/DDR-alterations and PFS. Swimmer plot of progression-free survival analysis according to specific HR-/DDR-genes and baseline clinicopathological characteristics. Line numbers refer to patient ID. Abbreviations: ICC, intrahepatic cholangiocarcinoma; ECC, extrahepatic cholangiocarcinoma; GBD, gallbladder tumor; 1L, first-line; ≥2L, second-line or above; CIS, cisplatin; GEM, gemcitabine; CHT, chemotherapy; IO, immunotherapy; DFI, disease-free interval.

Among 9 patients in the HRD-cohort demonstrating disease progression at the first radiological reassessment, gene alterations were found in ARID1A (*n* = 4), ATM (*n* = 1), BAP1 plus ARID1A (*n* = 1), BRCA2 (*n* = 1), NBN (*n* = 1), ATM (*n* = 1), and RAD51 (*n* = 1). Analysis of PFS according to the HRD genes is shown in Figure 4.

In the univariable Cox-regression analysis, the line of platinum chemotherapy (1L vs ≥2L) (HR 0.28, 95% CI, 0.10-0.80, *P* = .018), receipt of platinum regimen in the early setting (HR 5.21, 95% CI 1.92-14.1, *P* = .001), and disease-free interval (DFI,≥6 months vs <6 months, HR 0.05, 95%CI 0.01-0.41, *P* = .005) statistically associated with PFS (Supplementary Figures S2 and S3). In the multivariable model adjusting for variables associated with PFS, no difference was observed in PFS according to the HRD status (*P* = .5), while patients showing a disease-free interval above 6 months following curative-intent treatment for the early disease demonstrated better PFS (HR 0.06, 95% CI 0.10-0.50, *P* = .009) (Supplementary Figure S2).

OS events were observed for 56% (14 of 25) and 55.1% (27 of 49) of patients in the HRD-like and non-HRD group, respectively. No difference in median OS was observed between the HRD group (mOS 26.7 months, 95% CI, 19.4 to not evaluable [NE]) and non-HRD group (mOS 18.0 months, 95% CI, 14.9-32.5; HR, 0.67; 95% CI, 0.33-1.36, *Log-rank P* = .27) (Figure 3).

In the univariable Cox-regression model, only gallbladder tumors significantly associated with inferior OS (HR 2.76, 95% CI, 1.06-7.17, *P* = .038) (Supplementary Figures S1 and  S3), which were found to be more represented in the non-HRD group (11 of 49, 22% vs 3 of 25, 12%). Adjusting the main effect of the HRD status for tumor site, still, no difference emerged in OS between the HRD-like and non-HRD group (*P* = .4) (Supplementary Figures S1 and S3). Analysis of PFS and OS according to the primary tumor site is reported in Supplementary Figure S4.

## Discussion

The treatment landscape of BTC is rapidly evolving. Novel therapeutic options, mainly represented by targeted therapies and immunotherapy, are yielding new treatment opportunities for BTC. Still, most of the patients are affected by BTC not exhibiting actionable molecular alterations, and immunotherapy may portend potential long-term toxicities when added to platinum-based chemotherapy. Therefore, the recognition of predictive biomarkers for platinum chemotherapy in BTC currently represents an unmet clinical need to further achieve treatment personalization.

Accordingly, in this retrospective study, we aimed to investigate whether alterations in HR-/DDR-genes could predict the efficacy of platinum-based chemotherapy, wherein we observed limited clinical validity to predict long-term clinical outcomes.

In contrast to our analysis, a retrospective study involving 88 BTC demonstrated superior mPFS (6.9 months vs 5.7 months, *P* = .013) to platinum-chemotherapy for BTC showing alterations in HR-/DDR-genes.^22^ Similarly, among 150 intrahepatic BTC, tumors harboring HR-/DDR-alterations showed superior mPFS (7.3 months vs 5.4 months, *P* = .025). Conversely, in our study, we observed no difference in mPFS (5.18 vs 6.04 months, *P* = .95) in the HRD-like group and non-HRD group, respectively. Likewise, we observed comparable DCR between the 2 groups (64.0% vs 73.5% in HRD-like vs non-HRD tumors, *P* = .4), which was similar to those reported in the study of Rimini and colleagues across HRD-like and non-HRD BTC (DCR of 77.8% vs 67.9%, respectively, *P* = .04).^23^

In our study, a similar proportion of patients in the HRD-like (24%) and non-HRD group (30.6%) received previous systemic chemotherapy. Moreover, among the non-HRD cohort, 5 patients had previously been exposed to platinum-containing chemotherapy, which was significantly associated with inferior mPFS in our analysis. In contrast, the proportion of patients receiving previous platinum-based chemotherapy was not reported in the previous studies, with possible differences which may have accounted to some extent with the different findings in our study.

Conversely, we observed a numerically higher mOS among HRD-like tumors compared to non-HRD tumors (26.7 vs 18.0 months, respectively, *P* = .27), which was consistent with the findings of Chae et al, in which a superior mOS was observed for tumors showing HR-/DDR-alterations (21.0 months vs 13.3 months, *P* = .009).^22^ While the numerically superior OS observed in the HRD-like group could in part be related to a lower proportion of gallbladder tumors among them, associated in our analysis with worse outcomes, as previously shown,^24^ to random error given the small sample size, or both, the lower proportion of gallbladder tumors across both groups compared to previous studies in similar settings could also have accounted for the higher mOS we observed compared to historic controls.^2,8,9^

In the largest sequencing cohort of 489 BTC, deleterious or suspected deleterious alterations in HR-/DDR-genes were reported in 34% of cases, occurring in ARID1A (19.2%), BAP1 (9.4%), ATM (5.5%), BRCA2 (3.8%), PALB2 (1.9%), and BRCA1 (1.7%).^25^ Similarly, in our cohort, 47% (*n* = 35) of tumors showed alterations in HR-/DDR-genes, with oncogenic and likely oncogenic alterations found 33% (*n* = 25) of the cases. Of note, we included only deleterious alterations in our HRD-like cohort, to portend a more robust methodological selection for HR-/DDR-tumors as no pathological review with molecular biologists was conducted for variants of unknown significance (VUS). In contrast, the HRD-like cohort included a higher proportion of cases in the studies by Chae et al (63.5%) and Rimini et al (50%), for whom no genomic variants selection criteria have been reported for HRD-like tumors, limiting the inter-study comparisons.^22,23^

Similarly to these latter,^22,23^ in our HRD-like cohort, ARID1A represented the most common altered HR-/DDR-gene (13%, 10 of 25). Instead, we found only 2 patients affected by tumors showing BRCA1/2 alterations, with them showing a PFS of 1.55 months (BRCA2) and 8.45 months (BRCA1) before experiencing disease progression. Conversely, the study of Rimini et al and Chae et al encompassed a higher proportion of BRCA1/2 altered tumors (*n* = 13, 18%, and *n* = 16, 18.2%, respectively). Yet, no difference in mPFS was observed by Rimini et al in tumors carrying BRCA1/2 alterations, not suggesting the notion a different proportion of BRCA1/2 altered tumors may have accounted for differences in clinical outcomes observed in our study.^23^

Instead, the comparable outcomes achieved with platinum-based chemotherapy in our study between HRD-like and non-HRD tumors may be related to drawbacks in the analytical definition of HRD tumors. Indeed, while the presence of deleterious alterations in HR-/DDR-genes has been used to classify HRD tumors in clinical trials testing the use of PARPi,^26,27^ HRD reflects a complex biological phenotype that could arise from different mechanisms not limited to genomic sequence variants, as in the case of epigenetic alterations (eg, BRCA1 promoter methylation), which may not be intercepted by regular NGS platforms.^22,28^ Moreover, despite not being routinely assessed in clinical practice, a critical emerging biomarker of HRD is represented by the biallelic loss of function (LoF) in HR-/DDR-genes.^29,30^ As for most tumor suppressor genes, HR-/DDR-genes require a double-hit event in both alleles to yield a loss of protein function.^31^ Indeed, tumors carrying biallelic BRCA1/2 LoF demonstrate a higher frequency of HRD (81%) compared to tumors with monoallelic LoF (22%).^28,32^ Of note, most NGS panels cannot distinguish allele-specific LoF without dedicated bioinformatic pipelines, and consequently, we could not discriminate biallelic from monoallelic alterations in our HRD-cohort.^33,34^

Moreover, patients in our study underwent tumor-only sequencing. The frequency of monoallelic vs biallelic LoF in HR-/DDR-genes differs according to the germline status of the genomic alteration and to the cancer type. Tumors carrying germline BRCA1/2 (gBRCA1/2) alterations display a higher frequency of biallelic LoF (75-86%) compared to somatic BRCA1/2 (sBRCA1/2) (48%-61%).^32,33^ Considering the tumor type, BRCA1/2-associated cancers, including breast, ovarian, prostate, and pancreatic cancer, demonstrate a higher fraction of biallelic LoF for gBRCA1/2 (90%) and sBRCA1/2 (81%) compared to non-BRCA-associated cancers (46.3% for gBRCA1/2, 25.4% for sBRCA1/2).^32,33^ Consequently, the assessment of the allele-specific status becomes crucial when analyzing HR-/DDR-genes, particularly among non-BRCA-associated cancers, whose lack in our study could have led to an enrichment in tumors carrying monoallelic HR-/DDR-genes alterations in our cohort.

Nevertheless, compared to other non-BRCA-associated tumors, BTC displays a high-frequency of biallelic LoF, both in the setting of gBRCA2 alterations (65%) and sBRCA1/2 (61%).^32^ Moreover, BTC carrying biallelic LoF in BRCA1/2 has been shown to yield among the highest magnitude of HRD-scores (odd ratio 21.5), which was confirmed also for other HR-/DDR-genes.^30,32^ As such, BTC represent a tumor type for which investigating the status of HR-/DDR-genes might yield significant rationale, if being studied for allele-specific variants.

In addition, limited data is available concerning the impact of HR-/DDR-genes beyond BRCA1/2 to contribute with an HRD phenotype, and to what extent they may influence either PARPi or platinum efficacy.^30^ Indeed, while ovarian tumors with HRD without BRCA1/2 alterations demonstrate benefits from PARP-i,^35^ benefits from the use of PARP-i have been shown to be inconsistent in both prostate and breast cancers carrying either germline or somatic HR-/DDR-gene alterations beyond BRCA1/2.^19,27^ Moreover, in the TNT trial, among breast cancers demonstrating HRD, carboplatin did not yield superior outcomes compared to docetaxel, compared to tumors carrying gBRCA1/2 alterations.^36^ As such, several factors concerning the biology of HRD tumors remain to be elucidated, as demonstrated by our results.

Besides deleterious genomic variants in HR-/DDR-genes, genomic pipelines able to identify HRD genomic scars have been developed.^37-40^ Of note, HRD genomic signatures have been demonstrated to provide clinical utility to identify tumors showing responsiveness to PARPi also in tumors not exhibiting HR-/DDR-alterations.^41^ Accordingly, the application and possibly integration of analytical pipelines able to discriminate HRD-genomic scars may increase the analytical sensitivity and even expand the subgroup of tumors sensitive to DNA-damaging agents, such as platinum salts. Nevertheless, despite the analytical validity of HRD testing has been shown across different primary tumors,^32^ clinical validity and utility have not been assessed to date besides ovarian cancer,^41,42^ thus limiting their current applicability outside the context of clinical trials.

We recognize our study presents some limitations. First, our study included a limited number of patients, which may account for limited statistical power to detect differences between treatment groups, if present. Second, the observational retrospective design could have led to selection bias. Yet, we included the consecutive cohort of patients affected by BTC for which a comprehensive NGS test was available if a platinum regimen was received in the metastatic setting. Finally, as previously reported, our study included NGS panels not able to discriminate between somatic vs germline, and biallelic vs monoallelic genomic alterations, and no integration with complementary methods for HRD was available in our analysis.

## Conclusion

Our findings do not support the assessment of alterations in HR-/DDR-genes to identify BTC as more likely to benefit from platinum-based chemotherapy. Although our results may simply reflect a lack of biological association between HRD and platinum therapy efficacy, we recognize they may underscore the necessity to attain better analytical validation to identify HRD tumors. The HRD phenotype in solid tumors involves a complex biological background, necessitating comprehensive analytical approaches to capture critical genomic mechanisms, which current sequencing platforms only partially address. As such, further research is needed to investigate potential biomarkers capable of identifying BTC more likely to benefit from platinum-chemotherapy, including a precise assessment of the HRD-phenotype, to attain treatment personalization and ultimately better long-term prognosis for patients affected by biliary tract cancer.

## Supplementary Material

oyae125_suppl_Supplementary_Material

## Data Availability

The data underlying this article will be shared on reasonable request to the corresponding author.
