# Supplementary material for: Deleterious alterations in homologous recombination repair genes and efficacy of platinum-based chemotherapy in biliary tract cancers
Source: Oncologist. 2024 Jun 1;29(8):707–15. doi: 10.1093/oncolo/oyae125 (PMC11299956; doi:10.1093/oncolo/oyae125)

Supplementary Figure 1. Univariable Cox regression model: Progression-free survival (A) and Overall survival (B).

**A. Progression-free survival**	**HR**	**95% CI**	**p-value**
Group			
HRD			
non-HRD	0.98	0.56, 1.72	>0.9
De novo stage IV			
No			
Yes	0.95	0.56, 1.61	0.9
Previous (neo)adjuvant chemotherapy			
No			
Yes	1.44	0.82, 2.53	0.2
Platinum regimen for early disease			
No			
Yes	5.21	1.92, 14.1	0.001
Disease-free interval			
<6 months			
≥6 months	0.05	0.01, 0.41	0.005
Primary tumor type			
Extrahepatic			
Gallbladder	2.08	0.95, 4.54	0.068
Intrahepatic	0.99	0.52, 1.88	>0.9
Nr. Metastatic sites			
≤2			
≥3	0.61	0.33, 1.10	0.10
Platinum regimen			
ciplastin, gemcitabine, durvalumab			
cisplatin, gemcitabine	1.49	0.46, 4.84	0.5
other platinum-containing chemotherapy	2.86	0.82, 10.0	0.10
Line of first platinum chemotherapy			
≥2L			
1L	0.28	0.10, 0.80	0.018

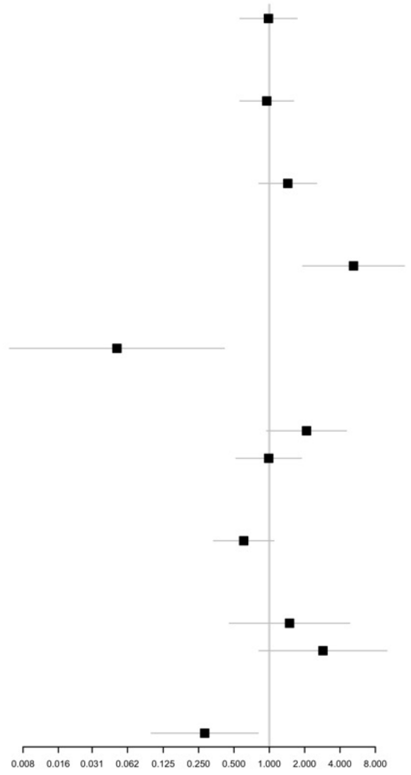

**B. Overall survival**	**HR**	**95% CI**	**p-value**
Group			
HRD			
non-HRD	1.49	0.73, 3.03	0.3
De novo stage IV			
No			
Yes	1.07	0.55, 2.08	0.8
Previous (neo)adjuvant chemotherapy			
No			
Yes	1.17	0.58, 2.36	0.7
Primary tumor type			
Extrahepatic			
Gallbladder	2.76	1.06, 7.17	0.038
Intrahepatic	0.72	0.34, 1.56	0.4
Nr. Metastatic sites			
≤2			
≥3	1.17	0.60, 2.27	0.6
Platinum_regimen			
ciplastin, gemcitabine, durvalumab			
cisplatin, gemcitabine	0.58	0.07, 4.55	0.6
other platinum-containing chemotherapy	0.66	0.08, 5.61	0.7

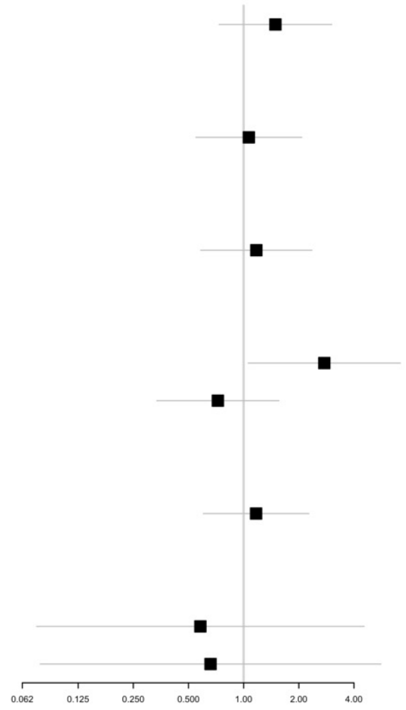

Supplementary Figure 2. Univariable and multivariable Cox regression model of PFS.

		Univariable			Multivariable		
**A. Progression-free survival**	**N**	**HR[1]**	**95% CI[1]**	**p-value**	**HR[1]**	**95% CI[1]**	**p-value**
Group	74						
HRD		—	—		—	—	
non-HRD		0.98	0.56, 1.72	>0.9	0.68	0.21, 2.20	0.5
De novo stage IV	74						
No		—	—				
Yes		0.95	0.56, 1.61	0.9			
Previous (neo)adjuvant chemotherapy	74						
No		—	—				
Yes		1.44	0.82, 2.53	0.2			
Platinum regimen for early disease	74						
No		—	—		—	—	
Yes		5.21	1.92, 14.1	0.001	2.76	0.49, 15.5	0.2
Disease-free interval	21						
<6 months		—	—		—	—	
≥6 months		0.05	0.01, 0.41	0.005	0.06	0.01, 0.50	0.009
Primary tumor type	74						
Extrahepatic		—	—				
Gallbladder		2.08	0.95, 4.54	0.068			
Intrahepatic		0.99	0.52, 1.88	>0.9			
Nr. Metastatic sites	74						
≤2		—	—				
≥3		0.61	0.33, 1.10	0.10			
Platinum regimen	74						
ciplastin, gemcitabine, durvalumab		—	—				
cisplatin, gemcitabine		1.49	0.46, 4.84	0.5			
other platinum-containing chemotherapy		2.86	0.82, 10.0	0.10			
Line of first platinum chemotherapy	74						
≥2L		—	—		—	—	
1L		0.28	0.10, 0.80	0.018	0.79	0.15, 4.29	0.8

[1] HR = Hazard Ratio, CI = Confidence Interval

Supplementary Figure 3. Univariable and multivariable Cox regression model of OS.

		Univariable			Multivariable		
**B. Overall survival**	**N**	**HR**[1]	**95% CI**[1]	**p-value**	**HR**[1]	**95% CI**[1]	**p-value**
Group	74						
HRD		—	—		—	—	
non-HRD		1.49	0.73, 3.03	0.3	1.42	0.68, 2.96	0.4
De novo stage IV	74						
No		—	—				
Yes		1.07	0.55, 2.08	0.8			
Previous (neo)adjuvant chemotherapy	74						
No		—	—				
Yes		1.17	0.58, 2.36	0.7			
Primary tumor type	74						
Extrahepatic		—	—		—	—	
Gallbladder		2.76	1.06, 7.17	0.038	2.96	1.12, 7.83	0.028
Intrahepatic		0.72	0.34, 1.56	0.4	0.80	0.36, 1.76	0.6
Nr. Metastatic sites	74						
≤2		—	—				
≥3		1.17	0.60, 2.27	0.6			
Platinum_regimen	74						
ciplastin, gemcitabine, durvalumab		—	—				
cisplatin, gemcitabine		0.58	0.07, 4.55	0.6			
other platinum-containing chemotherapy		0.66	0.08, 5.61	0.7			

[1] HR = Hazard Ratio, CI = Confidence Interval

Supplementary Figure 4. Analysis of PFS and OS according to the primary tumor site.

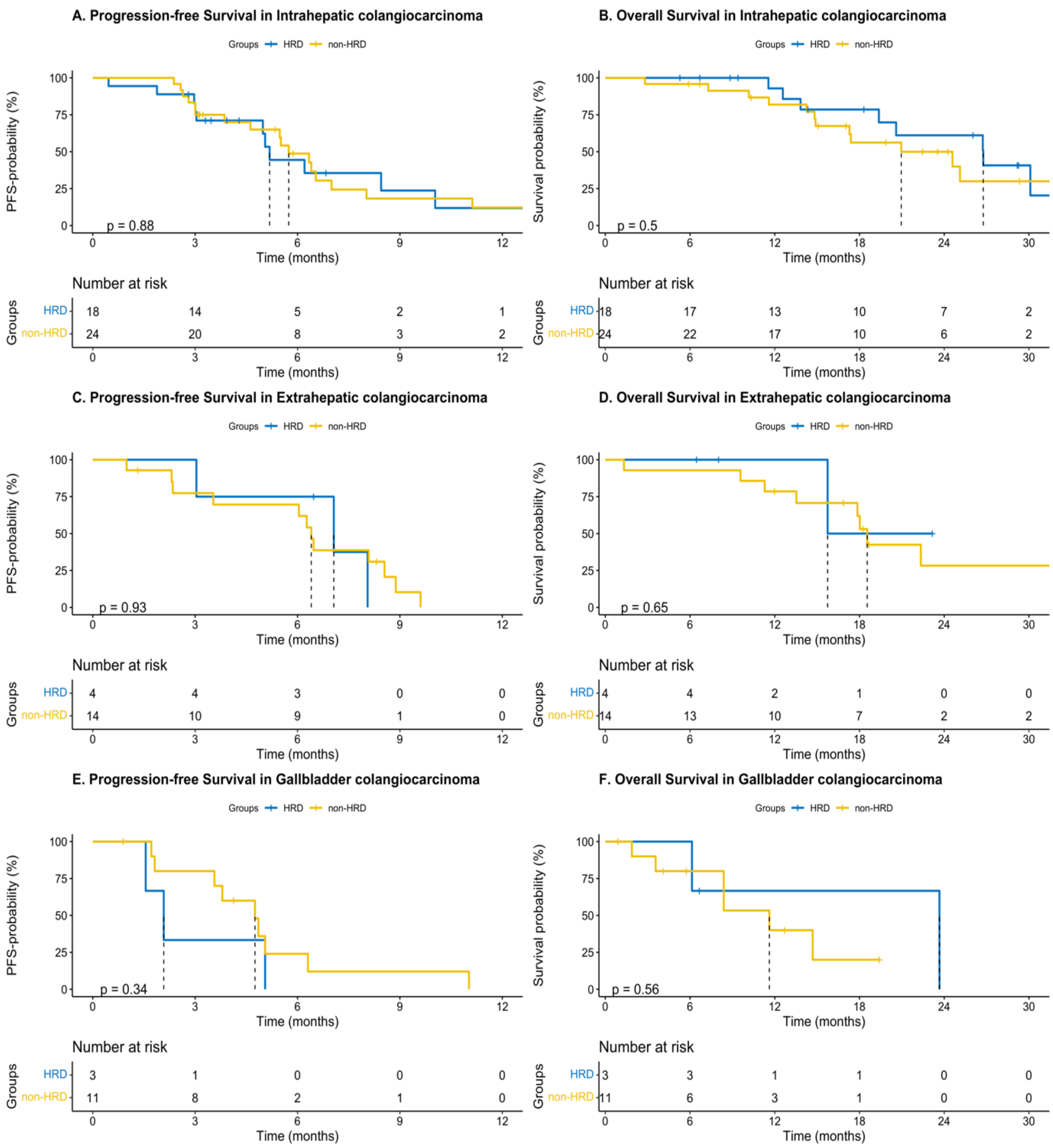

Supplement: oyae125_suppl_Supplementary_Material [file oyae125_suppl_supplementary_material.pdf]
